# Dietary factors and microRNA-binding site polymorphisms in the *IL13* gene: risk and prognosis analysis of colorectal cancer

**DOI:** 10.18632/oncotarget.17649

**Published:** 2017-05-07

**Authors:** Yanming Yu, Junde Zhou, Chen Gong, Zhiping Long, Jingshen Tian, Lin Zhu, Jing Li, Hongyuan Yu, Fan Wang, Yashuang Zhao

**Affiliations:** ^1^ Department of Epidemiology, School of Public Health, Harbin Medical University, Harbin, Heilongjiang Province, P. R. China; ^2^ Department of Colorectal Surgery, The Second Affiliated Hospital of Harbin Medical University, Harbin, Heilongjiang Province, P. R. China

**Keywords:** dietary factors, miRNA-binding site, polymorphisms, *IL13*, colorectal cancer

## Abstract

Long-term dietary intake influences the structure and activity of microorganisms residing in the human gut. The immune response and gut microbiota have a mutual influence on the risk of colorectal cancer (CRC). This study examines the association of gut microbiota–related dietary factors and polymorphisms in the microRNA-binding site of the interleukin 13 gene (*IL13*) with the risk and prognosis of CRC. Three polymorphisms (rs847, rs848, and rs1295685) were selected for genotyping in a case–control study (513 cases, 572 controls), and 386 CRC patients were followed up. Two dietary factors closely related with gut microbiota (allium vegetables, overnight meal) were significantly associated with CRC development. Although the three SNPs showed no statistically significant associations with the risk and prognosis of CRC, a significant antagonistic interaction was found between rs848 (G–T) and allium vegetable intake (ORi (odds ratio of interaction), 0.92; 95% CI (confidence interval): 0.86, 0.99; P = 0.03); moreover, significant combined and synergistic interactions were observed for all three SNPs and overnight meal intake. This is the first report of significant combined and interactive effects between dietary factors and polymorphisms in the microRNA binding site of *IL13* in CRC and may provide direct guidance on intake of allium vegetable and overnight meals for individuals with specific genetic variants of *IL13* to modify their susceptibility to CRC.

## INTRODUCTION

Colorectal cancer (CRC) is a major public health problem worldwide [1]. The World Health Organization reported that it is the third most common malignancy and the fourth most common cause of cancer mortality in the world in 2012. The incidence of CRC is higher in most developed countries but has been rapidly increasing in developing countries over recent years. There were 253,427 new cases and 139,416 deaths due to CRC in China in 2012 [2].

An increasing number of recent research studies have indicated that the gut microbiota is associated with a variety of diseases including obesity, inflammatory bowel disease, adenomas, and CRC [3–5]. Shen et al. [6] characterized the composition of adherent bacteria in normal rectal mucosal biopsies and observed that the gut bacterial composition of subjects with adenomas differed significantly from that of control subjects without adenomas. Brim et al. also noted a trend of altered microbial changes between adenoma patients and healthy controls by comparing the fecal microbiota [7]. Diet-induced changes to gut-associated microbial communities are now suspected to contribute to the growing epidemics of chronic illness [8–10]. Especially, food-borne microbes from the diet, including bacteria, fungi, and even viruses, transiently colonize the gut. In addition, high-throughput sequencing results revealed that inflammation modified the gut microbial composition only in colitis-susceptible interleukin-10-deficient (Il10^−/−^) mice [11]. Sears et al. [12] indicated that antibody-mediated blockade of IL-17 and the receptor for IL-23, a key cytokine that amplifies T-helper 17 cell responses, inhibits enterotoxigenic *Bacteroides fragilis*–induced colitis, colonic hyperplasia, and tumor formation. As dietary factors influence the structure and activity of the microorganisms residing in the human gut, inter-individual differences in colorectal cancer susceptibility may be mediated by the mutual influence of inflammatory gene expression and dysbiosis of gut microbiota. However, it is unclear how the human inflammatory genome interacts with dietary factors to affect colorectal carcinogenesis.

Interleukin 13 (IL-13) is an anti-inflammatory immunomodulatory cytokine that is produced by T and B cells, mast cells, and basophils. IL-13 inhibits the secretion of pro-inflammatory mediators such as prostaglandins, reactive oxygen species (ROS) and nitrogen species, tumor necrosis factor (TNF) alpha, and IL-1, -6, -8, and -12 [13]. Consequently, IL-13 exhibits anti-inflammatory and anti-tumor functions by eliciting the expression of activation-induced cytidine deaminase (AID), which can lead to the development of colitis and promote neoplastic transformation [14]. microRNAs (miRNAs) are endogenous non-coding RNAs of ~22 nucleotides (nts) that regulate gene expression in animals and plants by pairing with the 3′-untranslated regions (UTRs) of the messenger RNAs (mRNAs) of target genes and specifying mRNA cleavage or repression of protein synthesis [15]. Consistent with the important role of miRNAs in gene regulation, some 3′UTR polymorphisms in the vicinity of a miRNA binding site have been reported to interfere with miRNA function and lead to differential gene expression. Single nucleotide polymorphisms (SNPs) located within miRNA-binding sites could thus influence cancer risk and overall survival [16–18]. SNPs in the *IL13* gene have been reported to contribute to abnormal expression of IL-13 and modify susceptibility to cancer development [19]. However, the influence of SNPs in microRNA-binding sites of the *IL13* gene on the risk of colorectal cancer and overall survival has not been reported.

In this study we explored the association of dietary factors and polymorphisms in the microRNA-binding site of *IL13* with the risk and prognosis of CRC with the aim of providing meaningful instructions on dietary intake for individuals with specific genetic variants of *IL13*.

## RESULTS

In Supplementary Table 1 we present all the SNPs with minor allele frequency (MAF) > 5% located at the miRNA binding sites of genes involved in inflammatory processes. The sum of all |ΔΔG| values for each SNP was listed as the basis for the selection of SNPs in our study. Among SNPs located in microRNA binding sites, two SNPs (rs847, rs848) in the *IL13* 3′UTR had the highest values of |ΔΔG tot|, therefore we decided to examine polymorphisms in *IL13* in this study.

Table 1 shows the distribution of demographic characteristics for cases and controls and the baseline characteristics of cancer patients. A total of 513 CRC patients and 576 controls were recruited in this study. The mean age was 60.14 years for cases and 57.16 years for controls (*P* < 0.001). There was a higher proportion of workers with mental occupations and a lower proportion of physical workers among cases compared with controls (*P* = 0.003). The mean body mass index was 23.26 ± 3.35 and 24.27±4.14 in cases and controls, respectively (*P* < 0.001). No significant difference was found for the distribution of gender (*P* = 0.805), education level (*P* = 0.424), and family history of cancer (*P* = 0.168) between cases and controls.

**Table 1:** Demographics and baseline characteristics of study subjects

Characteristics	Cases (513)	Controls (576)	*P*- value
Age(years)^a^ mean ± s.d.	60.14 ± 11.29	57.16 ± 11.25	< 0.001
Gender			0.805
Male	302 (58.87)	334 (57.99)	
Female	211 (41.13)	242 (42.01)	
BMI(kg/m^2^)^a^ mean ± s.d.	23.26 ± 3.35	24.27 ± 4.14	< 0.001
Occupation			< 0.001
Mental worker	199 (38.79)	156 (27.08)	
Physical worker	224 (43.66)	266 (46.18)	0.003
Combined	86 (16.76)	154 (26.74)	
Education			0.424
Illiterate	134 (26.12)	168 (29.17)	
Primary school	151 (29.43)	174 (30.21)	
high school or above	204 (39.77)	210 (36.46)	
Family history of cancer			0.168
No	408 (79.53)	484 (84.03)	
Yes	94 (18.32)	89 (15.45)	
Tumor site			
Colon	168 (32.75)		
Rectal	312 (60.82)		
Cecum	30 (5.85)		

^a^Age and BMI are continuous variables, the others are categorical variables.

Missing data: tumor site, 3.

Based on multivariate logistic regression analysis for the association of dietary factors and CRC risk, cereals, vegetables, and milk, had protective roles whereas excessive consumption of pork, soybean, and fish braised in soy sauce were risk factors for CRC (detailed results are shown in Supplementary Table 2). Within the multivariate model, two dietary factors (allium vegetables and overnight meal) were found to be significantly associated with CRC development.

Table 2 shows the genotype distributions of three SNPs in *IL13* and their odds ratios (ORs) and 95% confidence intervals (CIs) for the risk of CRC. Age, BMI, and occupation were calculated as adjusted factors in the following analyses. Genotype distributions among controls were in agreement with the Hardy–Weinberg equilibrium. The frequencies of AA, AG, and GG genotypes of rs847 were 48.09%, 42.71%, and 8.51% in controls and 48.15%, 15.03%, and 6.04% in cases, respectively. The genotype frequencies for rs848 were 48.26% for GG, 41.67% for GT, and 8.50% for TT among controls and 48.15% for GG, 44.25% for GT, and 6.82% for TT among cases. None of the variant alleles was associated with the risk of CRC. Similarly, no significant association was observed between rs1295685 and the risk of CRC.

**Table 2:** Association between microRNA-binding site polymorphisms in *IL13* gene and the risk of colorectal cancer

Genotypes	Cases	Controls	OR_adjusted_ (95% CI)	*P*-value
	No.(%)	No.(%)		
rs847 A>G				
GG	247 (48.15)	277 (48.09)	1.00	
AG	231 (45.03)	246 (42.71)	0.69 (0.42–1.14)	0.15
AA	31 (6.04)	49 (8.51)	1.03 (0.80–1.33)	0.84
Dominant model			0.97 (0.76–1.25)	0.83
Recessive model			0.68 (0.42–1.11)	0.12
rs848 T>G				
GG	247 (48.15)	278 (48.26)	1.00	
GT	227 (44.25)	240 (41.67)	1.03 (0.79–1.33)	0.84
TT	35 (6.82)	49 (8.50)	0.78 (0.48–1.26)	0.31
Dominant model			0.99 (0.77–1.27)	0.93
Recessive model			0.77 (0.48–1.23)	0.27
rs1295685 C>T				
CC	245 (47.76)	272 (47.22)	1.00	
CT	225 (43.86)	238 (41.32)	1.02 (0.79–1.32)	0.87
TT	31 (6.04)	53 (9.20)	0.63 (0.39–1.04)	0.07
Dominant model			0.96 (0.75–1.22)	0.72
Recessive model			0.63 (0.39–1.01)	0.06

OR_adjusted_: adjusted for BMI, occupation, and age

As shown in Table 3, for allium vegetables, among individuals carrying the GG genotype of rs847 those with intake of allium vegetables (including green onion, garlic, onion) 4–6 times per week showed a statistically reduced risk of CRC compared with those with intake less than once per week (OR_dietary_ (OR_d_), 0.51; 95% CI: 0.30, 0.88; *P* = 0.02). The same association was observed among individuals carrying the GG genotype of rs848 (OR_d_, 0.53; 95% CI: 0.31, 0.91; *P* = 0.02) and in the analyses of rs1295685. However, we did not find significant results for individuals who consumed allium vegetables > 7 times/week.

**Table 3:** Combined and interactive effects of microRNA–binding site polymorphisms in IL13 and dietary factors on the risk of colorectal cancer

SNPs	Dietary factors	Cases (No.)	Controls (No.)	Adjusted OR_dg_ (95% CI)	P– value	Adjusted ORi (95% CI)	*P*– value*
rs847	Allium vegetables^#^ (times/week)					0.92 (0.84–1.01)	0.08
GG	< 1	83	69	1			
GG	1–3	59	68	0.85 (0.52–1.39)	0.53		
GG	4–6	34	59	0.51 (0.30–0.88)	0.02		
GG	> 7	71	79	0.81 (0.51–1.29)	0.37		
AG+AA	< 1	70	64	0.92 (0.57–1.49)	0.74		
AG+AA	1–3	61	75	0.68 (0.42–1.10)	0.11		
AG+AA	4–6	50	68	0.65 (0.39–1.08)	0.09		
AG+AA	> 7	80	88	0.83 (0.53–1.32)	0.44		
rs847	Overnight meal^$^ (times/week)					1.25 (1.08–1.43)	0.002
GG	< 1	47	64	1			
GG	1–3	96	121	1.11 (0.69–1.79)	0.68		
GG	> 3	104	92	1.66 (1.02–2.70)	0.04		
AG+AA	< 1	54	63	1.14 (0.67–1.97)	0.63		
AG+AA	1–3	96	142	0.90 (0.56–1.45)	0.67		
AG+AA	> 3	111	88	1.84 (1.13–3.01)	0.01		
rs848	Allium vegetables (times/week)					0.92 (0.86–0.99)	0.03
GG	< 1	84	69	1			
GG	1–3	57	70	0.79 (0.48–1.29)	0.34		
GG	4–6	35	57	0.53 (0.31–0.91)	0.02		
GG	> 7	71	80	0.78 (0.49–1.24)	0.29		
GT+TT	< 1	68	63	0.89 (0.55–1.44)	0.63		
GT+TT	1–3	63	70	0.72 (0.44–1.17)	0.18		
GT+TT	4–6	49	67	0.63 (0.38–1.05)	0.07		
GT+TT	> 7	81	89	0.81 (0.52–1.27)	0.36		
rs848	Overnight meal (times/week)					1.24 (1.08–1.42)	0.002
GG	< 1	49	63	1			
GG	1–3	97	120	1.07 (0.66–1.72)	0.79		
GG	> 3	101	95	1.41 (0.91–2.39)	0.12		
GT+TT	< 1	54	63	1.07 (0.62–1.83)	0.81		
GT+TT	1–3	93	139	0.84 (0.52–1.34)	0.45		
GT+TT	> 3	114	85	1.82 (1.12–2.96)	0.02		
rs1295685	Allium vegetables (times/week)					0.92 (0.84–1.00)	0.06
CC	< 1	84	67	1			
CC	1–3	58	66	0.82 (0.50–1.34)	0.42		
CC	4–6	34	58	0.50 (0.29–0.86)	0.01		
CC	> 7	69	79	0.76 (0.41–1.21)	0.24		
CT+TT	< 1	67	65	0.84 (0.52–1.36)	0.47		
CT+TT	1–3	59	73	0.64 (0.40–1.05)	0.08		
CT+TT	4–6	50	65	0.65 (0.39–1.07)	0.09		
CT+TT	> 7	79	88	0.78 (0.50–1.24)	0.29		
rs1295685	Overnight meal (times/week)					1.21 (1.05–1.39)	0.008
CC	< 1	46	63	1			
CC	1–3	96	121	1.12 (0.69–1.81)	0.65		
CC	> 3	103	88	1.72 (1.05–2.81)	0.03		
CT+TT	< 1	53	62	1.13 (0.65–1.95)	0.66		
CT+TT	1–3	92	138	0.90 (0.55–1.45)	0.65		
CT+TT	> 3	110	89	1.82 (1.16–2.98)	0.02		

*P value for interaction analysis conducted by multivariate logistic regression.

^#^Allium vegetables, vegetables in the *Allium* genus include onions, shallots, leeks and scallions, as well as herbs like garlic and chives.

^$^Overnight meal, the vegetables, egg, meat that have been cooked and left overninght.

SNP, Single Nucleotide Polymorphism

OR_dg_, OR for combined effects of dietary factors and genetic factors

OR_i_, OR for interactive effects of dietary factors and genetic factors

For overnight meal, among individuals carrying the GG genotype of rs847, those with intake of overnight meal > 3 times/week showed a significantly increased risk of CRC compared with those with less than one intake per week (OR_d_, 1.66; 95% CI: 1.02, 2.70; *P* = 0.04). Similar results were obtained among individuals carrying the CC genotype of rs1295685 (OR_d_, 1.72; 95%CI: 1.05, 2.81; *P* = 0.03). These concordant results indicate that overnight meal is a risk factor for CRC.

Combined effects (altered susceptibility to CRC because of co-exposure to genetic variation and dietary intake) and interactive effects (how the two different genotypes respond to environmental variation in different ways) were analyzed for these three microRNA binding site polymorphisms and two dietary factors. For allium vegetables, we found a statistically significant antagonistic interaction for rs848 (G–T) and allium vegetable intake (OR_interactiv_e [OR_i_]), 0.92; 95% CI: 0.86, 0.99; *P* = 0.03), and marginally significant interactive effects between rs847 (*P* = 0.08) or rs1295685 (*P* = 0.06) and allium vegetable intake. However, no significant combined effect was found for rs848 (G–T) and allium vegetable intake.

Compared with individuals carrying the GG genotype of rs847 and with < 1 time/week intake of overnight meal, those carrying the AG or AA genotypes and with more than 3 times/week intake of overnight meal showed a increased risk of CRC (OR_dietary&genetic_ [OR_dg_]), 1.84; 95% CI: 1.13, 3.01; *P* = 0.01). Especially, a significant synergistic interaction was observed between rs847 (G–A) and overnight meal, indicating that overnight meal and rs847 jointly increase the risk of CRC. Similar results were found for the association of rs848 or rs1295685 and overnight meal with the risk of CRC; significant combined effects were observed for overnight meal and rs848 (OR_dg_, 1.82; 95% CI: 1.12, 2.96; *P* = 0.02) and overnight meal and rs1295685 (OR_dg_, 1.82; 95% CI: 1.16, 2.98; *P* = 0.02). Corresponding synergistic effects were also significant for overnight meal and rs848 (OR_i_, 1.24; 95% CI: 1.08, 1.42; *P* = 0.002) and overnight meal and rs1295685 (OR_i_, 1.21; 95% CI: 1.05, 1.39; *P* = 0.008).

We analyzed the correlation between these three polymorphisms and clinical characteristics; however, no significant results were found (data are shown in Supplementary Table 3). Supplementary Table 4 shows the hazard ratio (HRs) and 95% CIs from univariate and multivariate Cox regression. Only general classification and Duke's stage remained significant in multivariate analysis. Compared with patients with protruding type of CRC, those with invasive and ulcer types showed shorter survival times and an increased risk of death (HR, 1.73; 95% CI: 1.23, 2.44). In addition, Dukes’ stage showed significance as a prognostic predictor; mean survival times of the patients decreased with an increase in Dukes’ stage. Compared with patients with Dukes’ stage I, the HR increased significantly for the other stages (Dukes’ stage II, HR, 1.60; 95% CI, 0.71, 3.61; Dukes’ stage III, HR, 4.29; 95% CI, 1.95, 9.42; Dukes’ stage IV, HR, 14.79; 95% CI, 5.97, 36.66).

## DISCUSSION

We first explored the associations between gut microbiota–related dietary factors (allium vegetables, overnight meal), polymorphisms in miRNA-binding sites of the *IL13* gene, and the risk of CRC. A key novel finding of this study was evidence for combined and interactive effects of SNPs in *IL13* and dietary factors in CRC development. The lack of association between *IL13* polymorphisms and overall survival or clinical pathological characteristics is unsurprising given that the altered gut microbiota caused by dietary factors and genetic variants in *IL13* may play a lead role in carcinogenesis rather than prognosis of CRC.

Several studies have reported the influence of SNPs in *IL13* on the risk of cancer, and a meta-analysis concluded that *IL13* rs20541 polymorphisms contribute to susceptibility to cancer [19]. In addition, growing evidence has indicated that genetic variants in the sequences of miRNA-binding sites could affect miRNA regulation to target gene expression and consequently modify susceptibility and the prognosis of several cancers [20–23]. Based on bioinformatics analysis, Mark et al. found an increased risk in both bladder and breast cancer for the homozygote variant of the *PARP-1* SNP rs8679 [24]. Landi et al. examined the association between SNPs in miRNA-binding regions and sporadic colorectal cancer risk and showed statistical significance of variant alleles of CD86 [25]. Another study conducted by Pan et al. reported that the let-7–targeted KRAS rs712 polymorphism was associated with an increased risk of colorectal cancer and may play crucial roles in the etiology of CRC [26]. rs848 included in our study was predicted to be located within the binding sites of miR-558, miR-621, and let-7i with total |ΔΔG|s of 156.5 kJ/mol. Similarly, rs847 and rs1295685 were predicted to have total |ΔΔG|s of 112.9 and 26.1 (Supplementary Table 1). However, we did not find any significant association of these three SNPs with the risk of colorectal cancer.

With respect to the functional capacity of adenoma- or carcinoma-related gut microbe(s), dietary factors and chronic inflammatory factors have been recognized as crucial causes of CRC [27–30]. Several previous studies have suggested that dietary habits play a key role in modulation of the gut microbiota composition [31, 32]. The impact of diet on the human gut microbiota is an important environmental factor in the pathogenesis of disease states such as inflammatory bowel diseases [33, 34]. Garlic contains oil-soluble organosulfur compounds such as ajoene, diallyl sulfide, diallyl disulfide, and diallyltrisulfide, whereas onion contains mainly S-propenylcysteinesulfoxide but also other sulfoxides [35]. Researchers found that diallyl sulfide can penetrate the membrane of bacteria [36]. Previous studies have validated that volatile thiosulfinates, unstable and volatile bioactive sulfur-containing compounds, have antimicrobial activity against *Helicobacter pylori* [37]. Xiaonan et al. [38] studied the effect of garlic compounds on the food-borne bacterium *Campylobacter jejuni*, the most prevalent cause of food poisoning worldwide, and showed that garlic-derived compounds can curb growth of food-borne *C. jejuni*. Thus, researchers suggested that garlic-derived organosulfur compounds have the potential to be used as antimicrobial agents [39]. Several epidemiological studies also showed an inverse association between the intake frequency of onion or garlic and the risk of several common cancers [3, 40, 41]. In this study, we observed that intake of allium vegetables 4, 5, 6 times per week could significantly reduce the risk of CRC. Moreover, we found significant antagonistic interactions between the *IL13* polymorphisms and intake of allium vegetables, indicating that the protective effects of allium vegetable intake were reduced in individuals with the variant allele of these three SNPs compared with individuals with wild-type genotype. However, overconsumption of garlic may stimulate the intestinal tract, causing intestinal mucosal hyperemia and edema aggravation. Based on the results of our study, intake frequency of more than 7 times/week for garlic or other allium vegetable is not recommended.

A recent study demonstrated that bacterial diversity is remarkably decreased in the gut microbiota of mice models of sporadic colorectal cancer and colitis-associated cancer [42]. Bacterial toxins in overnight meals could cause destruction of the normal gut microbial ecosystem and induce chronic gastroenteritis [3]. Moreover, a high level of nitrite is generated when bacteria multiply rapidly if the food storage method is incorrect. Nitrate and nitrite are precursors in the endogenous formation of potentially carcinogenic N-nitroso compounds (NOC). The Shanghai Women's Health study suggested that high dietary nitrate and nitrite intake results in increased exposure to endogenously formed NOCs and increased risk of CRC [43]. In this study, we found a significantly increased risk of CRC for individuals carrying genetic variants of all three SNPs combined with a high frequency of intake of overnight meals. The statistically significant synergistic interactions among the SNPs and overnight meal intake indicated that genetic variants and overnight meal collectively increased the susceptibility to CRC. These results highlighted the carcinogenic effects of overnight meals especially in individuals carrying variant alleles of these three SNPs, and suggest that long-term consumption of overnight meals should be avoided in such cases.

The role of polymorphisms in miRNA-binding sites as prognostic biomarkers and their correlations with the response to chemotherapy has been researched. Chae et al. [44] found that CRC patients carrying a variant allele of rs1044129 (miRNA-367 binding site) showed poor recurrence-free-survival compared with those with the AA or AG genotype. However, Kjersem et al. did not find a significant effect of SNPs in the let-7 microRNA binding site in KRAS (rs61764370) on progression-free survival and overall survival in patients receiving Nordic FLOX+cetuximab in the NORDIC-VII trial (NCT00145314) [45]. In the present study, no significant correlations between the different genotypes of the three SNPs of *IL13* and clinical pathological characteristics were observed. Similarly, there was no statistically significant effect of the three SNPs on overall survival of CRC patients.

There were some limitations in this study. First, recall bias may be inevitable in the collection of information on dietary factors, although we did our best to minimize this bias. Second, we only investigated three microRNA binding site polymorphisms in *IL13* rather than all inflammatory genes. Third, the bioinformatics strategy that we used for screening of microRNA-binding site polymorphisms may not be powerful enough to find genetic variants with the greatest biological impact. Although our study has a relatively large sample size, the number of individuals in some subgroups with variant homozygotes is still too small to obtain sufficient statistical power.

In summary, this is the first study using population epidemiological methods to elucidate the role of gut microbiota–related dietary factors and polymorphisms in miRNA-binding site in *IL13* in CRC. Although the three SNPs selected by screening using bioinformatics tools did not show significant independent associations with the development and prognosis of CRC, we observed significant combined and interactive effects between these three SNPs and dietary intake of allium vegetables and overnight meal. Future guidelines for dietary intake based on individual genetic background should be addressed.

## MATERIALS AND METHODS

### Study subjects

We performed this study after obtaining informed written consent from study subjects and approval from the Human Research and Ethics Committee of Harbin Medical University. All experiments were performed in accordance with relevant guidelines and regulations.

A case–control study was designed to assess the role of genetic polymorphisms and dietary factors on the risk of CRC. Cases were incident patients who underwent surgery at the Cancer Hospital and the Second Affiliated Hospital of Harbin Medical University from June 2004 to January 2008. Patients with neuroendocrine carcinoma, malignant melanoma, non-Hodgkin's lymphoma, gastrointestinal stromal tumors, and Lynch syndrome colorectal cancer were excluded. A total of 513 CRC patients with pathologic diagnosis were recruited. Controls were enrolled from patients in the orthopedic and ophthalmology departments who were admitted to the Second Affiliated Hospital of Harbin Medical University and volunteers from the Xiangfang community of Harbin city within the same time period. Any individual with a history of polyps, adenomas, or other disease related to cancer was excluded from controls. In total, 576 controls (77 community-based and 499 hospital-based) were recruited.

A patient cohort study was proposed to explore the potential factors associated with the prognosis of CRC. Among the 513 CRC patients, 386 were followed up from November 2004 to March 2014 with telephone interview.

All subjects in this study were informed and gave written consent to participate in the study. All procedures, including participant recruitment, questionnaire information collection, and all experimental protocols, were approved by the Human Research and Ethics Committee of Harbin Medical University.

### Data collection

The questionnaire is structured to collect information on demographic characteristics (age, gender, height and weight, education, marital status, occupation, and race) and dietary factors relevant to CRC development. For each subject, history of smoking and drinking, detailed disease history and family history of cancer, and dietary status during the past 1 year before cancer diagnosis were recalled. Peripheral venous blood was obtained and stored at −80°C immediately after separation of plasma. DNA was extracted from blood samples of 513 cases and 576 controls using the classic phenol–chloroform procedure [46] and QIAamp DNA Blood mini kits (Hilden, Germany).

Clinical information including tumor size, Duke's stage, chemotherapy, histological and pathological types, and serum levels of carcinoembryonic antigen (CEA) and carbohydrate antigen 19-9 (CA19-9) before surgery were extracted from medical records. Overall survival (OS) was calculated from the first day of cancer diagnosis to death. Patients who suffered from recurrence and were still alive at the end of follow-up were measured as censored data.

### SNP selection and genotyping

We initially analyzed 49 candidate genes involved in inflammatory processes. All SNPs residing on miRNA binding sites within the 3′UTRs were captured by an extensive search in dbSMR(https://omictools.com/dbsmr-tool). Of these, 21 genes were selected according to minor allele frequency of the Chinese population > 5% in Pubmed (Supplementary Table 1). Using RNA hybrid (http://bibiserv.techfak.uni-bielefeld.de/rnahybrid/submission.html), the Gibbs free energy (DG, expressed in kilojoules per mole [kJ/mol]) for both wild-type and variant alleles of each identified SNP was determined and the difference in DG between the two alleles (wild-type allele DG−variant allele DG) was calculated as ΔΔG. The sum (|ΔΔG tot|) of all |ΔΔG| values for each SNP was calculated as a parameter for predicting the biological impact of the polymorphism.

The fluorogenic 5′-nuclease assay (TaqMan SNP Genotyping Assay, Applied Biosystems, Foster City, CA, USA) was used to analyze genomic DNA samples for *IL13* polymorphisms. Analysis was performed using the Roche Lightcycler 480II Sequence Detection System. The 25-μl reaction mix contained at least 10 ng DNA, 12.5 μl Universal PCR Master Mix, and 0.625 μl Probe/Primer mix. The assay ID numbers of *IL13* were as follows: rs847: C_8932046_10; rs848: C_8932051_20; rs1295685: C_8932052_10. PCR amplification conditions were an initial step of 95°C for 10 min followed by 40 cycles of 92°C for 15 s and 60°C for 1 min. The genotyping experiment was conducted according to the protocol of the TaqMan^®^ Assay.

### Statistical analysis

Each polymorphism was tested to confirm fit with Hardy–Weinberg equilibrium with alpha threshold of 0.05 for controls. Categorical and continuous variables were tested by chi-square test and two-sample t test respectively. Univariate and multivariate logistic-regression analyses were used to calculate crude and adjusted odds ratios (ORs) and 95% confidence intervals (CIs) for the association of *IL13* microRNA binding site SNPs and CRC risk. In multivariate analysis, significant variables from univariate analysis were selected and manually entered into the model step by step. The combined and interactive effects between genetic variants and dietary factors were estimated by crossover study and multivariate logistic regression. The cutoff of *P*-values was 0.05 in both univariate and multivariate analyses.

Kaplan–Meier curves and log-rank test were used to assess the influence of *IL13* variants on overall survival. Hazard ratios (HRs) and corresponding 95% confidence intervals (CIs) were computed using univariate and multivariate Cox proportional hazard models. Data were analyzed using SPSS 17.0 software, and all *P*-values represent two-sided statistical tests.

## SUPPLEMENTARY MATERIALS TABLES









## Abbreviations

CRC: colorectal cancer
IL-17: interleukin-17
IL-13: interleukin 13
TNF: tumor necrosis factor
AID: activation-induced cytidine deaminase
miRNAs: microRNAs
mRNAs: messenger RNAs
Nts: nucleotides
UTRs: untranslated regions
SNPs: Single Nucleotide Polymorphisms
OR_i_: OR_interactive_
OR_dg_: OR_dietary&genetic_
NOC: N-nitroso compounds
RFS: recurrence-free-survival
CEA: carcinoembryonic antigen
CA19-9: carbohydrate antigen 19-9
OS: overall survival
ORs: odds ratios
CIs: confidence intervals
HRs: Hazard Ratios.
