# Supplementary material for: Dietary factors and microRNA-binding site polymorphisms in the IL13 gene: risk and prognosis analysis of colorectal cancer
Source: Oncotarget. 2017 May 7;8(29):47379–88. doi: 10.18632/oncotarget.17649 (PMC5564572; doi:10.18632/oncotarget.17649)
Supplement: Supplementary file 1 [file oncotarget-08-47379-s001.pdf]

# Dietary factors and microRNA-binding site polymorphisms in the *IL13* gene: risk and prognosis analysis of colorectal cancer

## Supplementary Materials

**Supplementary Table 1: The predicted biological impact of candidate SNPs located in microRNA-binding site of *IL13*.** See Supplementary_Table_1

**Supplementary Table 2: Univariate and multivariate logistic regression analyses for the association between environmental factors and colorectal cancer risk.** See Supplementary_Table_2

**Supplementary Table 3: Demographic and clinical pathological characteristics of colorectal cancer patients.** See Supplementary_Table_3

**Supplementary Table 4: Cox-regression on the association between clinical pathological characteristics, microRNA-binding site polymorphisms in *IL13* and the prognosis of CRC**

| Prognosis factor | Univariate analysis | | Multivariate analysis | |
|---|---|---|---|---|
| | Mean survival times (months) (95% CI) | Hazard ratio (HR) (95% CI) | Hazard ratio (HR) (95% CI) | *P* value |
| **Location of primary tumor** | | | | |
| Colon | 77.17 (68.84–85.51) | 1 | | |
| Rectum | 70.18 (64.81–75.55) | 1.37 (0.94–2.00) | | |
| **General classification of tumor** | | | | |
| Protrude type | 81.08 (73.58–86.57) | 1 | 1 | |
| Invasive and ulcerative type | 61.70 (54.10–69.30) | 2.02 (1.44–2.84) | 1.73 (1.23–2.44) | 0.002 |
| Other types | 11.50 (8.04–14.97) | 9.83 (1.32–73.23) | 14.92 (1.95–14.47) | 0.009 |
| **Histological classification of tumor** | | | | |
| Adenocarcinoma | 71.33 (66.22–76.43) | 1 | | |
| Mucinous adenocarcinoma | 75.75 (65.52–85.98) | 0.86 (0.56–1.33) | | |
| Other types | 80.17 (51.38–108.95) | 0.55 (0.14–2.23) | | |
| **Stage of Dukes'** | | | | |
| I | 92.44 (83.81–101.07) | 1 | 1 | |
| II | 85.27 (79.30–91.23) | 1.67 (0.78–3.54) | 1.60 (0.71–3.61) | 0.26 |
| III | 59.70 (51.71–66.43) | 4.28 (2.06–8.90) | 4.29 (1.95–9.42) | < 0.001 |
| IV | 22.35 (15.05–29.65) | 15.72 (6.78–36.48) | 14.79 (5.97–36.66) | < 0.001 |
| **Metastasis** | | | | |
| No | 54.52 (47.72–61.32) | 1 | | |
| Yes | 86.29 (81.12–91.47) | 3.11 (2.21–4.37) | | |
| **Chemotherapy treatment** | | | | |
| No | 73.85 (67.28–80.41) | 1 | | |
| Yes | 72.11 (65.86–78.36) | 0.96 (0.69–1.34) | | |
| **rs847** | | | | |
| GG | 67.82 (61.39–74.26) | 1 | | |
| AG | 76.86 (70.13–83.60) | 0.72 (0.51–1.01) | | |
| AA | 83.18 (65.97–100.38) | 0.51 (0.22–1.17) | | |
| **rs848** | | | | |
| GG | 67.91 (61.48–74.35) | 1 | | |
| GT | 76.08 (69.32–82.84) | 0.75 (0.53–1.06) | | |
| TT | 78.80 (60.49–97.11) | 0.60 (0.28–1.30) | | |
| **rs1295685** | | | | |
| CC | 66.94 (60.46–73.43) | 1 | | |
| CT | 77.37 ( (70.59–84.14) | 0.69 (0.49–0.97) | | |
| TT | 84.57 (68.08–101.06) | 0.47 (0.20–1.07) | | |
