# Supplementary material for: Dietary factors and microRNA-binding site polymorphisms in the IL13 gene: risk and prognosis analysis of colorectal cancer
Source: Oncotarget. 2017 May 7;8(29):47379–88. doi: 10.18632/oncotarget.17649 (PMC5564572; doi:10.18632/oncotarget.17649)
Supplement: Supplementary file 2 [file oncotarget-08-47379-s002.docx]

**Supplemental table 1** The predicted biological impact of candidate SNPs located in microRNA-binding site of *IL13*

| Gene, dbSNP ID and  allele substitution^a^ |  | MAF^b^ | Predicted binding miRNA |  | ΔΔG^c^ |  | \|ΔΔG_tot_\|^d^ |
| --- | --- | --- | --- | --- | --- | --- | --- |
| *IL12* RB2 rs1874396 A>G |  | 0.149 |  |  | 26.0 |  | 26.0 |
| *IL13* rs1295685 C>T |  | 0.240 | mir621 |  | 26.1 |  | 26.1 |
| *IL13* rs848 T>G |  | 0.350 | mir558 |  | 27.3 |  | 156.5 |
|  |  |  | mir595 |  | 18.3 |  |  |
|  |  |  | mir-621 |  | 14.7 |  |  |
|  |  |  | mir-632 |  | 21.2 |  |  |
|  |  |  | let-7i |  | 17.1 |  |  |
|  |  |  | let-7d |  | 13.8 |  |  |
|  |  |  | let-7f |  | 12.5 |  |  |
|  |  |  | let-7g |  | 17.1 |  |  |
|  |  |  | mir-98 |  | 14.5 |  |  |
| *IL13* rs847 A>G |  | 0.246 | mir-98 |  | 23.5 |  | 112.9 |
|  |  |  | let-7i |  | 28.8 |  |  |
|  |  |  | let-7f |  | 29.9 |  |  |
|  |  |  | let-7g |  | 30.7 |  |  |
| *IL13RA1* rs2495636 A>G |  | 0.274 | mir-129 |  | 27.2 |  | 27.2 |
| *IL15RA* rs2296135 G>T |  | 0.461 | mir-767 |  | 13.4 |  | 29.4 |
|  |  |  | mir-184 |  | 16.0 |  |  |
| *IL16* rs859 C>T |  | 0.494 | mir-612 |  | 36.4 |  | 36.4 |
| *IL16* rs11325 T>G |  | 0.243 | mir-612 |  | 36.4 |  | 75.2 |
|  |  |  | mir-147 |  | 24.6 |  |  |
|  |  |  | mir-640 |  | 14.2 |  |  |
| *IL17RB* rs3017 T>C |  | 0.398 | mir-7 |  | 26.3 |  | 26.3 |
| *IL18RAP* rs7559479 A>G |  | 0.277 | mir-136 |  | 11.6 |  | 11.6 |
| *IL18RAP* rs7603250 A>G |  | NA | mir-136 |  | 20.4 |  | 20.4 |

**Supplemental table1** continue

| Gene, dbSNP ID and  allele substitution^a^ |  | MAF^b^ | Predicted binding miRNA |  | ΔΔG^c^ |  | \|ΔΔG_tot_\|^d^ |
| --- | --- | --- | --- | --- | --- | --- | --- |
| *IL19* rs1798 C>G |  | 0.184 | mir-654 |  | 27.0 |  | 27.0 |
| *IL36RN* rs768627 C>T |  | 0.329 | mir-647 |  | 29.8 |  | 29.8 |
| *IL1RL1* rs12905 C>T |  | 0.239 | mir-132 |  | 8.6 |  | 34.9 |
|  |  |  | mir-183 |  | 26.3 |  |  |
| *IL1RL1* rs12712142 A>C |  | 0.386 | mir-371 |  | 11.4 |  | 11.4 |
| *IL1RN* rs315951 G>C |  | 0.399 | mir-758 |  | 28.3 |  | 28.3 |
| *IL23R* rs10889677 G>T |  | 0.365 | mir-494 |  | 18.7 |  | 18.7 |
| *IL28RA* rs10903032 C>T |  | 0.497 | mir-105 |  | 23.6 |  | 49.6 |
|  |  |  | mir-512-3p |  | 26.0 |  |  |
| *IL28RA* rs10903034T>C |  | 0.496 | mir-105 |  | 23.6 |  | 49.6 |
|  |  |  | mir-512-3p |  | 26.0 |  |  |
| *IL28RA* rs8832 A>G |  | 0.438 | mir-338 |  | 26.0 |  | 26.0 |
| *IL8RB* rs1126579 C>T |  | 0.449 | mir-134 |  | 26.2 |  | 35.7 |
|  |  |  | mir-628 |  | 9.5 |  |  |

^a^SNPs located in *IL13* with predicted ΔΔG >3 kJ/mol and MAF>5%; ^b^MAF, minimum allele frequency; ^c^ΔΔG, DDG, difference of DG between the two alleles (wild-type allele DG – variant allele DG); ^d^|ΔΔG_tot_|, the sum of all the |ΔΔG|s. DG, the Gibbs free energy.
