# Supplementary material for: Dietary factors and microRNA-binding site polymorphisms in the IL13 gene: risk and prognosis analysis of colorectal cancer
Source: Oncotarget. 2017 May 7;8(29):47379–88. doi: 10.18632/oncotarget.17649 (PMC5564572; doi:10.18632/oncotarget.17649)
Supplement: Supplementary file 3 [file oncotarget-08-47379-s003.docx]

**Supplemental table 2** Univariate and multivariate logistic regression analyses for the association between environmental factors and colorectal cancer risk

| **Variables** | **Cases** | **Controls** |  | **Univariate analysis** | |  | **Multivariate analysis** | |
| --- | --- | --- | --- | --- | --- | --- | --- | --- |
|  | No.% | No.% |  | OR_crude_  (95% CI) | *P* value |  | OR_adjusted_  (95% CI) | *P* value |
| **Cereals (g/week)** |  |  |  |  |  |  |  |  |
| <50 | 245(47.85) | 172(30.18) |  | 1 |  |  | 1 |  |
| 50-100 | 114(22.27) | 133(23.33) |  | 0.56(0.40-0.78) | 0.001 |  | 0.51(0.33-0.79) | 0.003 |
| 100-200 | 70 (13.67) | 102(17.89) |  | 0.40(0.27-0.58) | <0.001 |  | 0.33(0.21-0.53) | <0.001 |
| >200 | 83 (16.21) | 163(28.60) |  | 0.30(0.21-0.42) | <0.001 |  | 0.29(0.19-0.45) | <0.001 |
| **Vegetables (g/day)** |  |  |  |  |  |  |  |  |
| <100 | 69 (13.50) | 46 (8.03) |  | 1 |  |  | 1 |  |
| ≥100 | 442(86.50) | 527(91.97) |  | 0.60(0.40-0.90) | 0.01 |  | 0.50(0.29-0.87) | 0.01 |
| **Fruits (times/week)** |  |  |  |  |  |  |  |  |
| 0 | 93 (18.13) | 89 (15.51) |  | 1 |  |  |  |  |
| <2 | 149(29.04) | 161(28.05) |  | 0.94(0.65-1.37) | 0.76 |  |  |  |
| 3-6 | 117(22.81) | 101(17.60) |  | 1.13(0.75-1.70) | 0.55 |  |  |  |
| ≥7 | 154(30.02) | 223(21.24) |  | 0.63(0.43-0.91) | 0.02 |  |  |  |
| **Type of meat** |  |  |  |  |  |  |  |  |
| None | 40 (7.91) | 32 (5.58) |  | 1 |  |  |  |  |
| Lean meat | 215(42.49) | 295(51.48) |  | 0.62(0.37-1.03) | 0.07 |  |  |  |
| Both | 220(43.48) | 207(36.13) |  | 0.90(0.54-1.52) | 0.70 |  |  |  |
| Fat meat | 31 (6.12) | 39 (6.81) |  | 0.68(0.34-1.34) | 0.26 |  |  |  |
| **Beef or mutton(g/week)** |  |  |  |  |  |  |  |  |
| 0 | 216(42.52) | 233(41.76) |  | 1 |  |  |  |  |
| <250 | 220(43.31) | 214(38.35) |  | 1.09(0.83-1.43) | 0.53 |  |  |  |
| ≥250 | 72 (14.17) | 111(19.89) |  | 0.77(0.53-1.11) | 0.17 |  |  |  |
| **Pork (g/week)** |  |  |  |  |  |  |  |  |
| 0 | 80 (15.94) | 91 (16.40) |  | 1 |  |  | 1 |  |
| <250 | 174(34.66) | 239(43.06) |  | 0.83(0.58-1.21) | 0.34 |  | 0.77(0.48-1.23) | 0.27 |
| 250-1000 | 188(37.45) | 195(35.14) |  | 1.16(0.80-1.69) | 0.44 |  | 1.01(0.63-1.64) | 0.96 |
| >1000 | 60 (11.95) | 30 (5.41) |  | 3.07(1.75-5.37) | <0.001 |  | 3.04(1.44-6.43) | 0.004 |
| **Poultry (g/week)** |  |  |  |  |  |  |  |  |
| 0 | 195(39.55) | 247(46.17) |  | 1 |  |  |  |  |
| <250 | 238(48.28) | 227(42.43) |  | 1.34(1.02-1.75) | 0.03 |  |  |  |
| ≥250 | 60 (12.17) | 61 (11.40) |  | 1.29(0.85-1.95) | 0.23 |  |  |  |
| **Freshwater fish (times/week)** |  |  |  |  |  |  |  |  |
| <1 | 206(40.31) | 246(42.78) |  | 1 |  |  |  |  |
| ≥1 | 305(59.69) | 329(57.22) |  | 1.11(0.87-1.43) | 0.40 |  |  |  |
| **Seafood (times/week)** |  |  |  |  |  |  |  |  |
| <1 | 398(83.97) | 447(87.99) |  | 1 |  |  |  |  |
| ≥1 | 76 (16.03) | 61 (12.01) |  | 1.40(0.96-2.05) | 0.08 |  |  |  |
| **Drinking water** |  |  |  |  |  |  |  |  |
| Well water | 72 (14.09) | 113(25.28) |  | 1 |  |  | 1 |  |
| Piped water | 419(82.00) | 294(65.77) |  | 2.39(1.66-3.44) | <0.001 |  | 2.15(1.38-3.34) | 0.001 |
| Purified water | 20 (3.91) | 40 (8.95) |  | 0.84(0.43-1.65) | 0.62 |  | 0.60(0.27-1.37) | 0.23 |

**Supplemental table 2** continue.

| **Variables** | **Cases** | **Controls** |  | **Univariate analysis** | |  | **Multivariate analysis** | |
| --- | --- | --- | --- | --- | --- | --- | --- | --- |
|  | No.% | No.% |  | OR_crude_  (95% CI) | *P* value |  | OR_adjusted_  (95% CI) | *P* value |
| **Soybean (times/week)** |  |  |  |  |  |  |  |  |
| 0 | 32 (6.27) | 54 (9.38) |  | 1 |  |  | 1 |  |
| ≤1 | 80 (15.69) | 100(17.36) |  | 1.42(0.82-2.44) | 0.21 |  | 2.08(0.99-4.37) | 0.05 |
| 2-3 | 182(35.69) | 259(44.97) |  | 1.12(0.69-1.83) | 0.65 |  | 1.39(0.71-2.73) | 0.34 |
| ≥4 | 216(42.35) | 163(28.29) |  | 2.18(1.33-3.58) | 0.002 |  | 3.10(1.55-6.18) | 0.001 |
| **Milk (times/week)** |  |  |  |  |  |  |  |  |
| 0 | 285(55.99) | 215(48.31) |  | 1 |  |  | 1 |  |
| ≤2 | 51 (10.02) | 71 (15.73) |  | 0.49(0.32-0.75) |  |  | 0.37(0.22-0.61) | <0.001 |
| ≥3 | 173(33.99) | 159(35.96) |  | 0.68(0.50-0.93) |  |  | 0.68(0.46-0.99) | 0.004 |
| **Tea** |  |  |  |  |  |  |  |  |
| No | 142(27.84) | 144(25.00) |  | 1 |  |  |  |  |
| Yes | 368(72.16) | 432(75.00) |  | 1.11(0.84-1.46) |  |  |  |  |
| **Fried food (times/month)** |  |  |  |  |  |  |  |  |
| ≤1 | 357(69.86) | 435(75.78) |  | 1 |  |  |  |  |
| 2-3 | 101(19.77) | 93 (16.20) |  | 1.32(0.95-1.82) |  |  |  |  |
| ≥4 | 53 (10.37) | 46 (8.02) |  | 1.44(0.94-2.23) |  |  |  |  |
| **Barbecue (times/month)** |  |  |  |  |  |  |  |  |
| ≤1 | 450(88.06) | 507(88.02) |  | 1 |  |  |  |  |
| 2-3 | 28 (5.48) | 37 (6.42) |  | 0.89(0.52-1.48) | 0.63 |  |  |  |
| ≥4 | 33 (6.46) | 32 (5.56) |  | 1.50(0.87-2.56) | 0.14 |  |  |  |
| **Allium vegetables (times/week)** |  |  |  |  |  |  |  |  |
| <1 | 154（30.08） | 133（23.17） |  | 1 |  |  | 1 |  |
| 1-3 | 120（23.44） | 144（25.09） |  | 0.77(0.55-1.09) | 0.14 |  | 0.69(0.44-1.09) | 0.11 |
| 4-6 | 84 （16.41） | 127（22.13） |  | 0.60(0.42-0.88) | 0.01 |  | 0.48(0.30-0.78) | 0.003 |
| 7 | 154（30.07） | 170（29.61） |  | 0.84(0.61-1.18) | 0.32 |  | 0.64(0.41-1.00) | 0.05 |
| **Overnight food (times/week)** |  |  |  |  |  |  |  |  |
| 0 | 103（20.12） | 128（22.30） |  | 1 |  |  | 1 |  |
| 1-3 | 193（37.70） | 263（45.82） |  | 0.92(0.66-1.28) | 0.63 |  | 0.81(0.53-1.26) | 0.36 |
| >3 | 216（42.18） | 183（31.88） |  | 1.59(1.13-2.23) | 0.01 |  | 1.70(1.08-2.67) | 0.02 |
| **Canned fruit** |  |  |  |  |  |  |  |  |
| No | 438（89.39） | 511（94.63） |  | 1 |  |  |  |  |
| Yes | 52 （10.61） | 29 （5.37） |  | 1.97(1.21-3.21) | 0.01 |  |  |  |
| **Canned meat** |  |  |  |  |  |  |  |  |
| No | 463（94.68） | 510（94.44） |  | 1 |  |  |  |  |
| Yes | 26 （5.32） | 30 （5.56） |  | 0.96(0.55-1.67) | 0.87 |  |  |  |
| **Fish braised in soy sauce (times/week)** |  |  |  |  |  |  |  |  |
| <1 | 321（63.31） | 419（74.56） |  | 1 |  |  | 1 |  |
| ≥1 | 186（36.69） | 143（25.44） |  | 1.74(1.33-2.28) | <0.001 |  | 2.36(1.64-3.41) | <0.001 |
| **Fried** [**pig's**](app:ds:pig's) [**intestines**](app:ds:intestines) **(times/week)** |  |  |  |  |  |  |  |  |
| No | 239（73.54） | 397（89.21） |  | 1 |  |  |  |  |
| Yes | 86 （26.46） | 48 （10.79） |  | 3.91(2.55-6.00) | <0.001 |  |  |  |
| **Alcohol drinking** |  |  |  |  |  |  |  |  |
| No | 194（60.06） | 251（56.40） |  | 1 |  |  |  |  |
| Yes | 129（39.94） | 194（43.60） |  | 0.83(0.61-1.14) | 0.24 |  |  |  |
| **Smoking** |  |  |  |  |  |  |  |  |
| No | 302（59.22） | 306（53.13） |  | 1 |  |  | 1 |  |
| Yes | 208（40.78） | 270（46.87） |  | 0.77(0.60-0.99) | 0.04 |  | 0.66(0.48-0.93) | 0.02 |
