# Supplementary material for: Dietary factors and microRNA-binding site polymorphisms in the IL13 gene: risk and prognosis analysis of colorectal cancer
Source: Oncotarget. 2017 May 7;8(29):47379–88. doi: 10.18632/oncotarget.17649 (PMC5564572; doi:10.18632/oncotarget.17649)
Supplement: Supplementary file 4 [file oncotarget-08-47379-s004.docx]

**Supplemental table 3** Demographic and clinical pathological characteristics of colorectal cancer patients

| **Characteristic** | **Total(%)** | **rs847** |  |  | ***P*** | **rs848** |  | |  | ***P*** | **rs1295685** |  |  | ***P*** |
| --- | --- | --- | --- | --- | --- | --- | --- | --- | --- | --- | --- | --- | --- | --- |
|  |  | No. of GG(%) | No. of AG(%) | No. of AA(%) | value | No. of GG(%) | No. of  GT(%) | | No. of TT(%) | value | No. of CC(%) | No. of CT(%) | No. of  TT(%) | value |
| **Location of primary tumor** | | | | | | | | | | | | | | |
| Colon | 91(29.45) | 50(31.85) | 36(27.27) | 5(27.78) | 0.71 | 50(31.65) | | 35(26.92) | 5(26.32) | 0.66 | 49(31.21) | 32(25.00) | 6(31.58) | 0.51 |
| Rectum | 218(70.55) | 107(68.15) | 96(72.73) | 13(72.22) |  | 108(68.35) | | 95(73.08) | 14(73.68) |  | 108(68.79) | 96(75.00) | 13(68.42) |  |
| **General classification of tumor** | | | | | | | | | | | | | | |
| Protrude type | 187(63.18) | 101(65.16) | 75(61.98) | 11(61.11) | 0.94 | 102(65.38) | | 73(60.83) | 11(61.11) | 0.89 | 100(64.94) | 73(61.34) | 12(63.16) | 0.93 |
| Invasive and ulcerative type | 107(36.15) | 53(34.19) | 45(37.19) | 7(38.89) |  | 53(33.97) | | 46(38.33) | 7(38.89) |  | 53(34.42) | 45(37.82) | 7(36.84) |  |
| Other types | 2(0.67) | 1(0.65) | 1(0.83) | 0 |  | 1(0.65) | | 1(0.84) | 0 |  | 1(0.64) | 1(0.84) | 0 |  |
| **Histological classification of tumor** | | | | | | | | | | | | | | |
| Adenocarcinoma | 244(78.96) | 125(79.62) | 104(78.79) | 13(72.22) | 0.67 | 126(79.75) | | 102(78.46) | 14(73.68) | 0.69 | 126(80.25) | 101((78.91) | 14(73.68) | 0.67 |
| Mucinous adenocarcinoma | 59(19.09) | 30(19.11) | 24((18.18) | 5(27.78) |  | 30((18.99) | | 24(18.46) | 5(26.32) |  | 29(18.47) | 23(17.97) | 5(26.32) |  |
| Other types | 6(1.95) | 2(1.27) | 4(3.03) | 0 |  | 2(1.26) | | 4(3.08) | 0 |  | 2(1.28) | 4(3.12) | 0 |  |
| **Stage of Dukes’** |  |  |  |  |  |  | |  |  |  |  |  |  |  |
| Ⅰ | 37(12.01) | 15(9.55) | 18(13.74) | 4(22.22) | 0.55 | 15(9.49) | | 18(13.95) | 4(21.05) | 0.55 | 15(9.55) | 18(14.17) | 4(21.05) | 0.55 |
| Ⅱ | 135(43.83) | 69(43.95) | 58(44.27) | 8(44.44) |  | 69(43.67) | | 56(43.41) | 9(47.37) |  | 69(43.95) | 54(42.52) | 9(47.37) |  |
| Ⅲ | 116(37.66) | 60(38.22) | 48(36.65) | 6(33.34) |  | 61(38.61) | | 48(37.21) | 6(31.58) |  | 60(38.22) | 48(30.57) | 6(31.58) |  |
| Ⅳ | 20(6.5) | 13(8.28) | 7(5.34) | 0 |  | 13(8.23) | | 7(5.43) | 0 |  | 13(8.28) | 7(12.74) | 0 |  |
| **Degree of differentiation** | |  |  |  |  |  | |  |  |  |  |  |  |  |
| Low | 49(15.86) | 34(21.66) | 15(11.36) | 0 | 0.12 | 34(21.52) | | 15(11.54) | 0 | 0.12 | 34(21.66) | 14(10.94) | 0 | 0.09 |
| Medium | 238(77.02) | 113(71.97) | 106(80.30) | 17(94.44) |  | 114(72.15) | | 104(80.00) | 18(94.74) |  | 113(71.97) | 103(80.47) | 18(94.74) |  |
| High | 7(2.27) | 3(1.91) | 4(3.03) | 0 |  | 3(1.90) | | 4(3.08) | 0 |  | 3(1.91) | 4(3.13) | 0 |  |
| Unknown | 15(4.85) | 7(4.46) | 7(5.30) | 1(5.56) |  | 7(4.43) | | 7(5.38) | 1(5.26) |  | 7(4.46) | 7(5.46) | 1(5.26) |  |
| **Metastasis** |  |  |  |  |  |  | |  |  |  |  |  |  |  |
| Yes | 135(43.69) | 74(47.13) | 53(40.15) | 6(33.33) | 0.33 | 75(47.47) | | 53(40.77) | 6(31.58) | 0.30 | 74(47.13) | 53(41.41) | 6(31.58) | 0.34 |
| No | 174(56.31) | 83(52.87) | 79(59.85) | 12(66.67) |  | 83(52.53) | | 77(59.23) | 13(68.42) |  | 83(52.87) | 75(58.59) | 13(68.42) |  |
| **Chemotherapy treatment** | |  |  |  |  |  | |  |  |  |  |  |  |  |
| Yes | 133(43.46) | 68(44.15) | 61(46.92) | 4(22.22) | 0.15 | 69(43.95) | | 58(45.31) | 4(21.05) | 0.13 | 68(43.59) | 59(46.83) | 4(21.05) | 0.11 |
| No | 173(56.54) | 88(55.85) | 69(53.08) | 14(77.78) |  | 88(56.05) | | 70(54.69) | 15(78.95) |  | 88(56.41) | 67(53.17) | 15(78.95) |  |
| **Anastomat on surgery** |  |  |  |  |  |  | |  |  |  |  |  |  |  |
| Yes | 220(72.37) | 111(71.61) | 95(73.64) | 13(72.22) | 0.70 | 111(71.15) | | 94(74.02) | 14(73.68) | 0.61 | 111(71.61) | 91(72.80) | 14(73.68) | 0.73 |
| No | 70(23.03) | 35(22.58) | 30(23.26) | 5(27.78) |  | 35(22.44) | | 29(22.83) | 5(26.32) |  | 35(22.58) | 30(24.00) | 5(26.32) |  |
| Undetermined | 14(4.6) | 9(5.81) | 4(3.1) | 0 |  | 10(6.41) | | 4(3.15) | 0 |  | 9(5.81) | 4(3.2) | 0 |  |
